# Seroprevalence of Herpes Simplex Virus Type 1 and 2 in Taiwan and Risk Factor Analysis, 2007

**DOI:** 10.1371/journal.pone.0134178

**Published:** 2015-08-07

**Authors:** Jen-Hsiang Shen, Kuan-Ying Arthur Huang, Chen Chao-Yu, Chih-Jung Chen, Tzou-Yien Lin, Yhu-Chering Huang

**Affiliations:** 1 College of Medicine, Chang Gung University, Taoyuan, Taiwan; 2 Division of Pediatric Infectious Disease, Department of Pediatrics, Chang Gung Memorial Hospital, Taoyuan, Taiwan; 3 Molecular Infectious Disease Research Centre, Chang Gung Memorial Hospital, Taoyuan, Taiwan; Wayne State University School of Medicine, UNITED STATES

## Abstract

**Background:**

Herpes simplex virus type 1 (HSV-1) and 2 (HSV-2) are common human pathogens and might cause severe illness. Following primary infection, the viruses establish lifelong latent infection and are transmitted by close contact, both sexual and nonsexual. However, the information about the seroprevalence of HSV-1 and HSV-2 across all age groups is limited.

**Methods:**

Residual sera collected during the nationwide serosurvey in 2007 in Taiwan were selected for the study. The enzyme-linked immunosorbent assay was used to detect anti-HSV-1 and anti-HSV-2 type-specific glycoprotein IgG. Demographics and personal health data were used for risk analysis.

**Results:**

A total of 1411 and 1072 serum samples were included for anti-HSV-1 and anti-HSV-2 seroprevalence analysis, respectively. The weighted overall seroprevalence was 63.2% for HSV-1, and 7.7% for HSV-2, respectively. The HSV-1 seropositive rate was 19.2% for those less than 5 years old, increased to 46.4% for those aged 5–13 years, 60.9% for those aged 14–29 years, and reached as much as 95.0% for those aged over 30 years. In contrast, the HSV-2 seropositve rate was 1.6% for those less than 30 years old, rose to 10.1% for those age 30–39 years, and was up to 31.2% for those aged over 60 years. A significantly higher HSV-2 seropositive rate was noted in females than males aged over 40 years (26.3% v.s. 16.8%), and the overall HSV-2 seropositive rate was almost twice higher in females than males. Smoking history, drinking habit, and educational level were associated with the HSV-1 seropositivity. Female gender and rural residence were independent factors for the HSV-2 seropositivity.

**Conclusions:**

An obvious increase of primary HSV-1 infection occurred in late adolescents and young adults, joined by the rise of HSV-2 infection in middle-aged adults, especially females. The acquistion and transmission of HSV warrant further studies in the susceptible population.

## Introduction

Herpes simplex virus type 1 (HSV-1) and type 2 (HSV-2), members of herpesviridae family, are common human pathogens. Most primary HSV-1 and HSV-2 infections are asymptomatic and self-limited, but can be complicated with fulminant diseases in neonates, young children, and immunocompromized hosts [1–9]. Traditionally, HSV-1 is considered as a pathogen of oral vesicular lesions. However, increasing evidence shows the emerging genital HSV-1 infections in adolescents and young adults [10, 11]. HSV-2 is a common pathogen of human sexually transmitted disease. Transmission of HSV from infected women to neonates may result in severe neurologic illness, disseminated disease, or death in the newborns [9–12]. Moreover, HSV ocular infection is believed to be one of the most common causes of corneal blindness in the developed countries [13].

The protective immune response against the HSV infection might involve virus-specific cytotoxic T cells and humoral immunity [14, 15]. Animal data showed the role of HSV-1-specific antibodies in limiting the disease severity [16]. The administration of the HSV-2 gD-2 vaccine conferred partial protection against primary HSV-1 infection in sero-negative women [17]. High HSV gD-2-specific antibody titer correlated to the efficacy of vaccine in the susceptible population [18]. Recently, the epidemiological data showed the decline of HSV-1 seropositive rate in adolescents and young adults in developed countries and this might warrant the increased risk of genital HSV infection while reaching the age of sexual debut [19].

Currently, there is no commercial vaccine available for prophylaxis and therapy of HSV infection in humans. Identifying the viral seroepidemiology within communities and applying proper public health measures would be important to control HSV infections. Here, we conducted a large-scale seroprevalence study to examine the HSV-1 and HSV-2 seropositive rates across all age groups and identify the factor associated with the seropositivity in Taiwan.

## Materials and Methods

### Study samples

In 2007, 3554 blood samples were collected from sixteen randomly selected administrative regions in Taiwan, representing 95.1% of the Taiwanese population, in one large public health survey [20–22]. Briefly, we used the age-stratified sampling design and invited individuals who visited local official health centers for routine immunization and/or health checkup to participate in the serosurvey [21, 22]. Blood samples were collected from enrolled subjects.

To examine the HSV-2 seroprevalence, 1,072 samples with residual serum were randomly selected from all age groups. We included 122 in 481 samples (25.4%) in the 0-4-year-old group, 71 in 310 (22.9%) in the 5-7-year-old group, 57 in 316 (18.0%) in the 8-10-year-old group, 55 in 276 (19.9%) in the 11-13-year-old group, 57 in 230 (24.8%) in the 14-16-year-old group, 78 in 322 (24.2%) in the 17-19-year-old group, 123 in 438 (28.1%) in the 20-29-year-old group, 129 in 258 (50.0%) in the 30-39-year-old group, 100 in 260 (38.5%) in the 40-49-year-old group, 91 in 260 (35.0%) in the 50-59-year-old group, 111 in 257 (43.2%) in the 60-69-year-old group, and 78 in 144 (54.2%) in the over 70-year-old group [21].

Regarding the HSV-1 seroprevalence study, since the seropositive rate was reported to be over 50% in the Chinese children at the age of five [23], we therefore included the majority of serum samples in the 0- to 4-year-old age group in the present study. In other age groups, the samples with residual sera were randomly selected, as previously described [21, 22].

The study was examined and approved by the ethics review committee of Chang Gung Memorial Hospital and all enrolled subjects/guardians provided written informed consent. In addition, questionnaires were used to collect the sociodemographic, lifestyle, and chronic medical information.

### Laboratory methods

The commercialized anti-HSV-1 (glycoprotein C1) and anti-HSV-2 (glycoprotein G2) enzyme-linked immunosorbent assay (ELISA) (IgG) kit (Euroimmun, Germany) were used to detect the antibody response in the serum samples.

Clinical serum samples from adult patients with laboratory-confirmed HSV infections were utilized to examine the sensitivity and specificity of the ELISA kit. The sensitivity and specificity of both ELISA kits were 100% and 100%, respectively, with a cut-off value of 22 relative units (RU)/ml. In addition, both the intra- and inter-assay coefficients of variability were less than 10% for anti-HSV-1 and anti-HSV-2 ELISA kits, respectively.

The analysis of anti-HSV-1 and anti-HSV-2 antibody responses was performed following the manufacturer’s instructions. Briefly, the serum was diluted 1:100 and loaded to the pre-coated microplates. After incubation at room temperature for 30 minutes, the plates were washed and incubated with peroxidase-conjugated anti-human IgG at room temperature for 30 minutes. Following further wash, the plates were developed with kit substrates at room temperature for 15 minutes. Positive (IgG, human), negative (IgG, human) controls, and calibration (IgG, human) standards were included on each plate. The optical densities of the reaction wells were measured at 450 nm. The standard curve was established by calibrators and used to calculate HSV-1 and HSV-2-specific antibody concentration. The seropositivity was defined as the virus-specific antibody serum concentration of ≥ 22 RU/ml.

### Statistics

Statistical analysis was performed using the SPSS statistical software package (2008 SPSS Inc, USA). The weighting overall seropositive rate and seropositive rate by gender were calculated with the utilization of the demographic information in the 2007 nationwide serosurvey [22]. Chi-square test and Fisher’s exact test were performed to analyze the significant difference in the seropositive rate.

Age, gender, sociodemographic and lifestyle factors, including place of residence, educational level, marital status, smoking history, and alcohol use have been associated with the HSV-1/HSV-2 seropositive rate in previous studies [24–26]. Odds ratio estimates and corresponding 95% confidence level, for the HSV-1 and HSV-2 seropositive rates, were obtained with multiple logistic regression, adjusting for potential risk factors (age, gender, rural/urban region, educational level, smoking history, drinking habit, marriage history, herpesviral seropositivity, and chronic medical conditions) in the univariate logistic regression analysis, as previously described [22]. P value < 0.05 was considered to be significant.

However, the information about sexual activities and behaviors, including age of sexual debut, ways of having sex, condom use during sex, numbers of sexual partners, history of sexual transmitted diseases, etc., was not collected in the serosurvey in 2007 and therefore not analyzed in the present study.

In the study, the definitions of rural/urban region, educational level, smoking history, drinking habit, smoking hisotry, and marriage history were previously described [22]. Briefly, the official definition about the population size and density per square kilometer was used to distinguish urban and rural regions in Taiwan, 2007. The level of education included ‘a graduate of university/college,’ ‘a graduate of high school,’ ‘a graduate of junior high school,’ ‘a graduate of elementary school,’ and ‘never been to the elementary school.’ The smoking history was classified as ‘non-smoker’ and ‘smoker.’ The drinking habit was classified as ‘less than 12 times in lifetime,’ ‘less than 12 times in one year,’ and ‘more than 12 times in one year.’ The marriage history was classified as ‘ever-married’ and ‘never-married.’

## Results

### The HSV-1 seroprevalence

**Fig 1:**
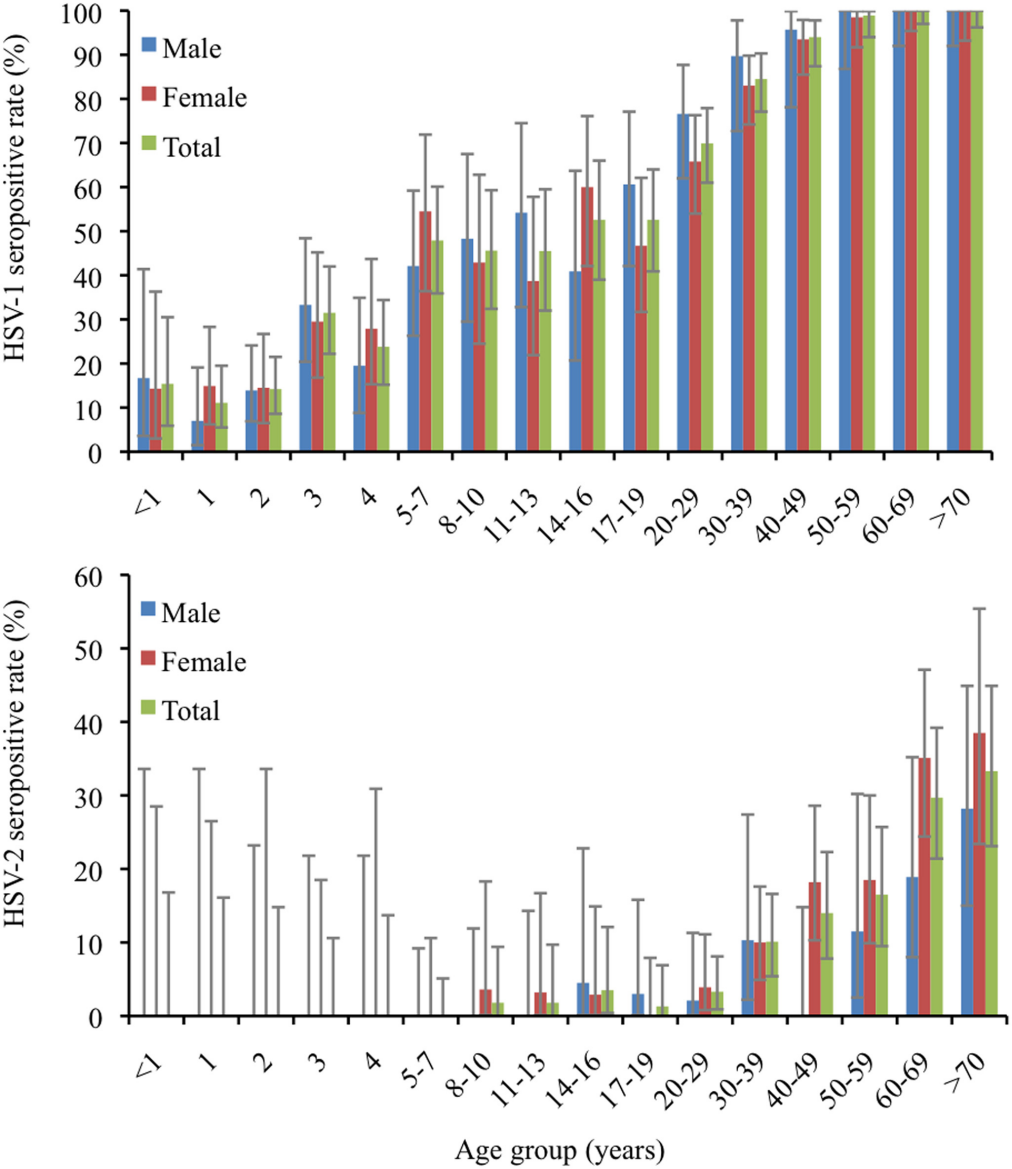
Age-specific and gender-specific seropositive rates of HSV-1 and HSV-2. Grey error bars represented 95% confidence intervals.

1411 serum samples were selected for analysis of the seroprevalence of HSV-1 in Taiwan, 2007. There were 830 females and 581 males. The age ranged from 2 months to 89 years old. The overall weighted seropositive rate of HSV-1 was 63.2% (95% CI, 60.6%-65.7%). Weighted seropositive rates were 64.5% (95% CI, 61.1%–67.7%) for women and 52.0% (95% CI, 47.8%–56.1%) for men, respectively. Infants had detectable HSV-1 IgG antibody level, which might be attributable to maternal antibody (Fig 1). In the early childhood, the HSV-1 seropositive rate increased from 11.1% at 1-year-old group to 47.9% at 5-7-year-old group. Until the early adolescent (age 11–13), the HSV-1 seropositive rate remained around 45%. Another significant rise of HSV-1 seropositive rate had been noted since late adolescent stage, from 53% at the 14–16 age group to over 90% at the 40–49 age group.

The association between the risk factor and the HSV-1 seropositivity was shown in Table 1. Age was significantly associated with the HSV-1 seropositivity (Table 1). Moreover, the logistic regression analysis showed that the drinking habit, smoking history, and the elementary school educational level accouted for the HSV-1 seropositivity. The HSV-2 seropositivity was also linked to an increased risk of HSV-1 seropositivity (adjusted odds ratio 10.64 with 95% CI 1.40–83.3, P = 0.022). No significant assoication between the gender and HSV-1 seropositivity was noted in the analysis. Neither the region of residence nor chronic illness was associated with the HSV-1 seropositivity.

**Table 1:** Risk factors for the HSV-1 seropositive rate.

	Age-adjusted OR (95% CI)	P value	Multivariate-adjusted OR (95% CI)	P value
Age			1.10 (1.07, 1.13)	<0.001
Female/Male	0.89 (0.67, 1.18)	0.420	1.06 (0.76, 1.46)	0.743
Rural/Urban	1.12 (0.73, 1.74)	0.594	1.03 (0.64, 1.67)	0.893
Ever-married/Never married	0.61 (0.34, 1.11)	0.103	0.67 (0.33, 1.36)	0.266
Smoker/Non-smoker	3.20 (1.43, 7.16)	0.005	3.25 (1.17, 9.05)	0.024
Drinking habit				
Less than 12 times in lifetime	1 (referent)		1 (referent)	
Less than 12 times per year	2.12 (1.26, 3.56)	0.004	2.15 (1.23, 3.77)	0.007
More than 12 times per year	2.04 (0.81, 5.10)	0.129	1.46 (0.51, 4.18)	0.477
Educational level				
Uneducated	1 (referent)		1 (referent)	
Elementary school	1.92 (1.21, 3.04)	0.005	2.13 (1.26, 3.62)	0.005
Junior high school	0.99 (0.54, 1.82)	0.967	1.50 (0.75, 3.00)	0.252
High school	0.75 (0.40, 1.38)	0.352	0.75 (0.37, 1.50)	0.412
College/University	0.58 (0.31, 1.08)	0.086	0.66 (0.33, 1.33)	0.247

Abbreviations: HSV-1, herpes simplex virus type 1; OR, odds ratio; CI, confidence intervals.

### The HSV-2 seroprevalence

We randomly selected 1,072 samples for the HSV-2 seroprevalence study. Among them, 663 (61.8%) were women. The mean age was 32.2 ± 23.3 years (range 0.2 ~ 89.8 years).

The overall weighted HSV-2 seropositive rate was 7.7% (95% CI, 6.2%–9.5%). The weighted seropositive rates among women and men were 12.1% (95% CI, 9.7%–14.8%), and 6.8% (95% CI, 4.6%–9.7%), respectively. Among those aged 0–7 years, none was positive for HSV-2 IgG antibody in the study. The HSV-2 seropositive rate in adolescents and young adults was between 1~3%. The seropositive rate reached 10% among those after 30 years old (Fig 1). Among middle-aged adults, women had significantly higher seropositive rate than men (P < 0.001). The overall HSV-2 prevalence was almost twice as high among women than men. The female gender was significantly associated with the HSV-2 seropositivity (Table 2).

**Table 2:** Risk factors for the HSV-2 seropositive rate.

	Age-adjusted OR (95% CI)	P value	Multivariate-adjusted OR (95% CI)	P value
Age			1.06 (1.04, 1.08)	<0.001
Female/Male	2.19 (1.34, 3.60)	0.002	3.15 (1.66, 5.96)	<0.001
Rural/Urban	1.72 (1.01, 2.91)	0.045	2.40 (1.37, 4.22)	0.002
Ever-married/Never married	0.58 (0.27, 1.27)	0.175	1.03 (0.45, 2.35)	0.946
Smoker/Non-smoker	1.41 (0.77, 2.59)	0.265	3.22 (1.54, 6.73)	0.002
Drinking habit				
Less than 12 times in lifetime	1 (referent)		1 (referent)	
Less than 12 times per year	0.77 (0.44, 1.33)	0.346	0.94 (0.52, 1.68)	0.826
More than 12 times per year	0.74 (0.36, 1.52)	0.414	1.00 (0.45, 2.22)	0.998
Educational level				
Uneducated	1 (referent)		1 (referent)	
Elementary school	0.55 (0.27, 1.13)	0.106	0.47 (0.21, 1.06)	0.070
Junior high school	0.51 (0.22, 1.16)	0.106	0.58 (0.24, 1.42)	0.233
High school	0.64 (0.33, 1.27)	0.201	0.72 (0.33, 1.56)	0.406
College/University	0.54 (0.27, 1.08)	0.082	0.61 (0.28, 1.33)	0.211

Abbreviations: HSV-2, herpes simplex virus type 2; OR, odds ratio; CI, confidence intervals.

In addition to age and gender, both the rural residence and HSV-1 seropositvity (adjusted odds ratio 12.82 with 95% CI 1.71–100.0, P = 0.013) were associated with the HSV-2 seropositivity. The smoking history seemed to be related with a higher risk of HSV-2 seropositivity. Neither the marriage history, education level, nor the drinking habit had statistical significance in relation to the HSV-2 seropositivity.

## Discussion

The study showed a similar overall seroprevalnce of HSV-1 as other large-scale studies conducted in Europe, which accounted for 60~70% in the population [27–29]. Although there was limited information about the HSV-1 seroprevalence across all age groups, especially in Asia, a regional study in eastern China showed a high overall HSV-1 seropositive rate, reaching 92% for those of 5 to 60-year-old [23]. The age-specific seropositive rate of HSV-1 could be as high as more than 80% among those over 30 years in this study and in China, Czech Republic, Bulgaria and Romania [23, 24, 29], but there observed a low seropositive rate in several developed countires, i.e. 61% in those aged 30–39 years in United States [19], around 40% in those aged 30–34 years in England and Wales [29], and around 50% in those aged 30–39 years in Japan [30]. The seropositve rates in childhood differed among regional studies, which ranged from 20% to 60% in the preschoolers [23, 27, 31, 32]. Children in the developing coutries seemed to have a higher HSV-1 seropositive rate than those in the developed countries.

The present study showed that the HSV-1 seropositive rate rose from 11% in 1-year-old children to 24% in children aged 4, and it doubled in children aged 5–7 in Taiwan. Non-sexual transmission might be the main route of acquistion of HSV-1 infection in young children. Close contact between young children (i.e. in day-care facilities or family members) and between adults and children were the most common way of spreading viruses between individuals. It was also suggested that hygiene and living conditions in childhood might play a role in the HSV-1 serostatus [13, 19]. Moreover, the HSV-1 seropositve rate continued to rise in adolescents and middle-aged adults. In addition to non-genital transmission, sexual activities might contribute to the HSV-1 infection in late adolescents and young adults. Although we are unable to determine the frequency of genitally acquired HSV-1 infections in the present study, the latest analyses have addressed the issue regarding the substantial burden of HSV-1 genital herpes in young adults in the developed countries [10, 11]. Public health hazards could arise along with this epidemiological situation. Firstly, genital herpes could be associate with an increased risk of HIV infections [33, 34]. Secondly, pregnant women who acquire primary genital herpes might transmit HSV-1 to newborns during labor and delivery and neonatal HSV infections have a high risk of severe morbidity [9]. In additonal to genital infection, the trend in the ocular infection rate could be associated with the age-specific HSV-1 seroprevalence [13]. Ongoing surveillance of the HSV-1 seroepidemiology and induction of the prospectivie follow-up study focusing on the natural history of viral acquistion would be important to clarify the rising trend of HSV-1 seropositivity and imporve public health strategies in the target populations.

The overall HSV-2 seropositive rate was around 10% in other large-scale studies in Asia [23, 30]. Several studies in the late twentieth century had shown 15% to 29% for the HSV-2 seropositive rate, but these studies mainly enrolled hospital-based samples or focused on adult women population [27]. The HSV-2 seroprevalence in Asia seemed to be lower than that in other geographic regions [27], i.e., 15% in USA [19], 17% in French [35], and even 60% in Zimbabwe [36], although variations in the cohort enrollment, the age distribution, or the test method could be found among these studies. A well-designed, cross-country study in the target population with identical serological methods might provide the comparable estimate of global HSV-2 prevalence. A recent report in the United States showed the fluctuations in the HSV-2 prevalence among those 14–49 years since 1970s, a significant rise was noted from 13.4% in 1976–1980 to 15.7% in 2005–2010, but no significant change was found between 1990s and 2000s [19]. However, the information about the time trends in the HSV-2 prevalence in a population scale has been lacking in Asia and further follow-up study should be conducted.

Several demographic factors, including gender [19, 37] and ethnicity [38], were associated with the HSV-1 seropositivity. Nevertheless, the difference in the HSV-1 seropositivity between men and women was not significant in the present and other studies [30, 35]. Among sociodemographic factors, lower education level was associated with the HSV-1 seropositivity in this study, which was compatible with the results in previous studies [24, 35, 39]. Besides, we noted that the rural residence was associated with the acquisition of HSV-2. In contrast, the rural residence could either be associated with lower HSV-2 seroprevalence [25, 37] or be an insignificant factor [39]; nevertheless, the definition of rural/urban region differed in these seroprevalence surveys. With regard to lifestyle factors, the drinking habit and smoking hisotry were associated with the HSV-1 seropositivity. This might be linked to the increased frequencey of close contacts and saliva exchanges during the drink and smoking activities. The smoking history was also a risk factor for the HSV-2 seropositivity [30, 40–42]. It was suggested that the cigarette and nicotine exposure might contriube to the immunosuppression and increase the susceptibility to respiratory viral infections [43], but the mechanism underlined the relationship between the smoking and the HSV infection remained largely unclear.

The present study clearly showed that female gender was associated with the HSV-2 seropositivity. This was also found in other HSV seroprevalence studies [27, 44, 45]. Several reasons have been proposed to explain the higher HSV-2 seropositivity in women than men, including the higher transmission rate of HSV-2 from men to women than it from women to men, the susceptibility of female genital mucosa to HSV-2 infection, and the association between hormone usage and genital herpes acquisition [46–48]. The acquisition of HSV-2 primarily results from sexual contacts. The genital lesions shed virus at high viral load in the symptomatic HSV-2 infection and reactivation and became the primary source of viral transmission [46, 49, 50]. Moreover, the majority of seropositive individuals might have subclinical viral shedding and this would facilitate the spread of HSV-2 during sexual activities. To reduce the burden of HSV-2 infections in the susceptible population, firstly, public health education about the recognition of herpes lesions should be promoted and this might raise awareness of risk of viral transmission and thus minimize high-risk sexual contacts. Secondly, using condoms during the sexual activity can be the alternatively effective way to reduce the likelihood of viral transmission [51]. Thirdly, with the introduction of reliable and sensitive PCR-based viral detection method, the timely implementation of antiviral therapy might be helpful to reduce the frequency of viral shedding and the risk of HSV-2 transmission as well [52–54].

Vaccine could be one of most powerful weapons against infectious disease in modern era. Considerable efforts have been made for the development of HSV-2 vaccine; however, the recent vaccine trial conducted in adult women has showed no significant efficacy against HSV-2 infection in immunized group [17].

The relationship between primary HSV-1 and HSV-2 infections has long been a debatable issue. The hypothesis that prior HSV-1 infection might either reduce the risk or decrease the clinical severity of HSV-2 infection was supported by the induction of cross-reactive cell-mediated immunity in the local tissues on the immunological basis [55]. Nevertheless, several large-scale, prospective, and longitudinal HSV epidemiological studies showed the controversial results [10, 39, 56]. Our results showed an association between HSV-1 and HSV-2 seropositivity, probably resulting from a substantial proportion of co-infection status. It has to be mentioned that we were unable to determine the time point of HSV acquisition and which type of HSV was precedently acquired from current data. A follow-up study including the viral isolation would be necessary to collaborate the findings found in the study.

In summary, this study demonstrated that there was an association of age factor with the HSV-1 seropositivity in the year 2007 in Taiwan. Young children, late adolescents, and young adults appeared to be susceptible populations with new HSV-1 infections. Moreover, there was a significant increase of HSV-2 seropositive rate in middle-aged adults, especially females. Continuing surveillance for the epidemiology, the route of transmission, and the implementation of public health strategies are highly recommended.

## Supporting Information

S1 Table The detailed data of age, gender, sociodemographic and lifestyle factors and HSV-1 and HSV-c2 serological results for enrolled subjects and the statistical data of the multivariate logistic regression for the HSV-1 and HSV-2 seropositivity. (XLS)
